# 

**Published:** 1903-09

**Authors:** 


*THE BRISTOL MEDICO-CHIRURGICAL SOCIETY.*

### CONTENTS.

| | PAGE |
|---|---|
| **Joseph Griffiths Swayne, M.D. (Lond.)** | 193 |
| **Round the World.** By R. ROXBURGH, M.B., F.R.C.S. Ed. | 202 |
| **A Contribution to the Conservative Surgery of the Uterine Appendages.** By JAMES SWAIN, M.S., M.D. Lond., F.R.C.S. | 211 |
| **A Case of Perforated Duodenal Ulcer, with Remarks.** By G. MUNRO SMITH | 218 |
| **The Antibodies in Disease.** By F. BUSHNELL, M.D. Lond. | 221 |
| **A Granuloma of the Nose, Due to Iodide of Potassium.** By C. PERCIVAL CROUCH, M.B. Lond., F.R.C.S. | 231 |
| **The Practical Value of Blood-Counts.** By WILLIAM GORDON, M.A., M.D. Cantab | 234 |
| **Four Cases of Renal Calculi Treated Successfully by Lumbar Nephro-Lithotomy.** By E. J. TREVOR JONES, M.D. Brux., M.R.C.S., L.R.C.P. Lond. | 245 |
| **Progress of the Medical Sciences:** | |
| **Medicine.** G. PARKER, M.D., M.A. Cantab. | 248 |
| **Surgery.** T. CARWARDINE, M.S., M.B. Lond., F.R.C.S. | 252 |
| **Electro-Therapeutics.** J. TAYLOR. | 256 |
| **Reviews of Books** | 260 |
| **Editorial Notes** | 279 |
| **Notes on Preparations for the Sick** | 281 |
| **The Library of the Bristol Medico-Chirurgical Society** | 284 |
| **Obituary Notices** | 285 |
| **Local Medical Notes** | 287 |

